# Short-chain fatty acids–microbiota crosstalk in the coronavirus disease (COVID-19)

**DOI:** 10.1007/s43440-022-00415-7

**Published:** 2022-09-27

**Authors:** Jakub Włodarczyk, Bartłomiej Czerwiński, Jakub Fichna

**Affiliations:** 1 Department of Biochemistry, Medical University of Lodz, Mazowiecka 6/8, 92-215 Lodz, Poland; 2 Department of General and Oncological Surgery, Medical University of Lodz, Pomorska 251, 92-215 Lodz, Poland

**Keywords:** COVID-19, SARS-CoV-2, SCFA, Probiotics, Microbiota

## Abstract

The novel coronavirus disease (COVID-19) still remains a major challenge to the health-care systems worldwide, inciting ongoing search for pharmaceutical and non-pharmaceutical interventions which could benefit patients already infected with SARS-CoV-2 or at increased risk thereof. Although SARS-CoV-2 primarily affects the respiratory system, it may also infect other organs and systems, including gastrointestinal tract, where it results in microbial dysbiosis. There is an emerging understanding of the role the gut microbiota plays in maintaining immune homeostasis, both inside the gastrointestinal tract and beyond (i.e. through gut–lung and gut–brain axes). One family of compounds with recognized immunomodulatory and anti-inflammatory properties are short chain fatty acids (SCFAs). SCFAs are believed that they have a protective effect in case of gastrointestinal diseases. Moreover, they are responsible for maintaining proper intestinal barrier and they take part in relevant immune functions. This review presents mechanisms of action and potential benefits of SCFA-based probiotics and direct SCFA supplementation as a strategy to support immune function amid the COVID-19 pandemic.

## Introduction

The novel coronavirus disease (COVID-19) caused by severe acute respiratory syndrome coronavirus 2 (SARS-CoV-2) primarily infects the respiratory system but also affects other organs including the gastrointestinal (GI) tract [1]. Although patients typically present with fever, dyspnea and cough, various non-specific symptoms reflecting multiorgan involvement may occur as well. COVID-related dysfunction in the GI tract may be present in a considerable percentage of patients—in some studies as high as 60%—and often manifests as diarrhea, nausea, vomiting and abdominal pain [2–5]**.** Notably, COVID-19 patients often test positive for SARS-CoV-2 RNA in their stool and viral genetic material can be detected in the feces even in the absence of GI symptoms, and its presence does not appear to be associated with the severity of illness [6]. Mostly, infected patients have a good prognosis. However, in some cases, the more severe course of disease is observed with many possible complications, including death. Established mortality risk factors are older age, male sex, presence of comorbidities (i.e. diabetes, cardiac or pulmonary disease, metabolic or immune disorders) [7].

SARS-CoV-2, similar to SARS-CoV, invades human cells with the use of angiotensin converting enzyme -2 (ACE2), a membrane protein expressed on multiple human cell types—which binds the viral spike protein [8, 9]. ACE2 expression has been evidenced in a multitude of different tissues around the body, with highest levels found in the small intestine, testis, kidneys, heart, thyroid, and adipose tissue, and moderate levels observed in the lungs, colon, liver, bladder, and adrenal gland. In the GI tract, ACE2 is present on the luminal surface of differentiated absorptive enterocytes in the small intestine and colon, but not on the goblet cells or intestinal immune cells [10, 11]**.** No significant difference between ACE2 expression levels in males and females, and between younger and older persons has been found [12]. ACE2 upregulation is, however, associated with smoking, diabetes, and cardiovascular diseases, all of which are linked with increased severity of and higher mortality from COVID-19[13]. However, in the literature, other secondary or potential receptors were identified as for SARS-CoV-2 entry. Studies suggest that AXL (tyrosine-protein kinase receptor UFO), KREMEN1 (Kringle-containing protein marking the eye and the nose protein 1) and ASGR1 (asialoglycoprotein receptor 1) may be involved in cell infiltration and multiple organ invasion [14, 15].

ACE2 is primarily associated with the renin–angiotensin system and regulation of blood pressure through vasoconstriction/vasodilation, but in the GI tract, ACE2 plays a significant role in amino acid homeostasis, innate immunity, and maintaining intestinal microbiota [10, 16]. ACE2 stabilizes neutral amino acid transporters, such as B(0)AT1, participates in degradation of digestive enzymes yielding free amino acids and also influences bacterial metabolism generating bioactive peptide fragments, including angiotensin II [17]. Loss of ACE2 in a knockout mouse model resulted in decline in the uptake of tryptophan—which plays an important role in immunity—and caused a decrease in the expression of antimicrobial peptides, and the change of the intestinal microbiota, which in turn resulted in high susceptibility to colitis [18]. High ACE2 expression in the GI tract may mediate the invasion of enterocytes by SARS-CoV-2, gut microbiome dysbiosis resulting in decreased SCFA production and activation of gastrointestinal inflammation, which is a possible mechanism of digestive symptoms in the COVID-19 patients [19, 20].

Dysbiosis can be defined as an imbalance of the gut’s microbiome, where a reduction in microbial diversity and loss of beneficial bacteria is observed [21]. Gut microbiomes are responsible for numerous vital functions such as promoting food digestion, stimulating the metabolism and regulating the innate and adaptive immunological processes [22]. Dysregulations in microbiome are linked with various diseases including inflammatory bowel diseases (IBD), irritable bowel syndrome (IBS), obesity, cancer, infectious diseases and central nervous system disorders [23]. However, up to date, no consensus conference has yet defined “dysbiosis”. Strong and constant fluctuations regarding age, gender, environmental conditions, diet, lifestyle make it troublesome to clearly define “dysbiosis” as well as “healthy microbiota” [24].

## Short-chain fatty acids

### Role of SCFAs in health and disease

Short-chain fatty acids (SCFA) are volatile fatty acids produced by the gut microbiota in the large bowel as fermentation products; they are characterized by containing fewer than six carbons and exist in straight and branched chain forms [25]. Acetate (C2), propionate (C3) and butyrate (C4) are the main SCFAs produced in the gut, but their proportion can vary greatly [26, 27]. Substrates for bacterial fermentation include non-digestible carbohydrates derived from dietary fibers such as polysaccharide plant cell walls, resistant starch, soluble oligosaccharide and endogenous products, such as mucin [27]. In the case of limited fiber supply, some bacterial species can switch to amino acids and protein fermentation, also contributing to SCFA and branched chain fatty acid production [28]. Fungi, such as *A. fumigatus*, can also produce SCFAs or create a biofilm enhancing the bacterial production of SCFAs, but their full impact on serum SCFAs levels has not been explored yet [29].

SCFAs produced in the gut may then enter the systemic circulation either by passive diffusion of undissociated SCFAs or, more importantly, by active transport using hydrogen- (MCT-1) and sodium-coupled (SMCT-1) transporters on epithelial cells [30]. Most SCFAs are then metabolized in the liver and only a limited fraction reaches the cardiovascular system. Acetate is the primary SCFA to enter circulation, and thus has the most potential to exert systemic anti-inflammatory effects [31]. In contrast, butyrate is primarily absorbed by colonocytes, which may explain why there were no significant changes in systemic inflammation in some studies that used butyrate supplementation. Circulating SCFAs act on a myriad of cells all around the body, modulating adipose tissue, skeletal muscle, pancreas and liver tissue function [32].

In the GI tract, SCFAs are not only a major energy source for intestinal cells, but also are involved in regulation of mucosal barrier integrity and immune homeostasis. SCFAs promote the development of naive CD4 + T cells into regulatory T cells [33], induce tolerogenic dendritic cells in the intestinal mucosa [34], and limit autoimmunological reactions [35]. They are also involved in promoting immune responses in the gut, such as inducing secretion of several interleukins, including IL-18 and IL-10 [36], and defensins (i.e. cathelicidin LL-37) [37]**,** inhibiting histone deacetylases (HDACs), and promoting epithelial cell repair [38]. Moreover, SCFAs in the gut decrease the luminal pH, curtailing growth of pathogenic bacteria and fungi [39]. Higher butyrate levels lead to increased mucin production which reduces bacterial adhesion and improves epithelial integrity [40], as well as to an increase in the number of Treg cells. SCFAs also influence the development of dendritic cells and inflammatory cytokines, thereby regulating the intestinal macrophages [41]. In particular, butyrate inhibits HDAC3, thus favoring macrophage polarization toward an anti-inflammatory M2 phenotype instead of pro-inflammatory M1 phenotype, which is typically more prevalent in case of ongoing viral infection [42].

SCFAs can be directly anti-inflammatory through the activation of peroxisome proliferator-activated receptor (PPAR)-γ, the suppression of NF-κB pathway, the inhibition of histone deacetylases, and the activation of G protein-coupled receptors (e.g., GPR41-FFAR3, GPR43-FFAR2, and GPR109a). Due to the presence of SCFAs in systemic circulation, this effect is not limited to the gut, as it was shown in the mouse model that butyrate treatment may also attenuate lung inflammation and mucus production by modulating Th9 cells [43]. Furthermore, butyrate was proven to have other pluripotential effects on multiple organs. Through inhibiting HDACs butyrate presents a beneficial role in atherosclerosis and vascular inflammation by decreasing the production of pro-inflammatory cytokines and restoring proper endothelial function [44]. Moreover, butyrate has inhibitory effect on NF-kB signaling pathway and stimulates the expression of anti-inflammatory cytokines (i.e. IL-10) in mononuclear cells and neutrophils [45]. SCFAs also improve kidney function by modulating inflammatory processes and limiting the effects of hypoxia after acute kidney injury by [46], which can develop in the context of SARS-CoV-2 infection.

Due to SCFAs involvement with the regulation of mucosal barrier integrity, deficiency of SCFAs is associated with an increased gut permeability, which allows the bacteria's translocation, triggering an inflammatory cascade [47], which could be a risk factor for worse outcome in COVID-19 patients. Noteworthy, Ohira et al. proved that butyrate downregulates genes crucial for SARS-CoV-2 infection such as Tmprss2 and Ace2, while increasing toll-like receptors (TLR) expression, which are involved in antiviral mechanisms [44]. Recent study by Takabayashi et al. (2021) showed that SCFAs effectively reduce the ACE2 levels in human airway epithelial cells in vitro [48]. If this property were to be observed in human intestinal cells, it would indicate that SCFAs may be limiting the virus’ ability to infect GI cells in COVID-19 patients.

It is worth mentioning that effects of SCFAs on viral replication rate have also been reported. One study demonstrated that butyrate increases cellular infection by H1N1 influenza A virus, reovirus and HIV-1 due to suppression of specific antiviral interferon-stimulated genes [49]. Another study demonstrated that mice fed a butyrate-rich diet had higher influenza virus titers during early stages of infection compared to control mice but experienced less tissue damage to lungs later in infection [50]. Taken together, these studies suggest that butyrate could reduce tissue damage caused by pro-inflammatory mechanisms involving type I IFN at the cost of increasing overall virus replicative capacity due to suppression of IFN-stimulated antiviral genes. In regard to SCFA impact on SARS-CoV-2 replication, there is one in vitro study, in which a mixture of acetate, propionate, and butyrate surprisingly did not alter the viral load of infected ex vivo human intestinal epithelial cells [51]. Some limitations of this study should be noted, such as the small sample size and the lack of intestinal biopsies from patients with COVID-19. Furthermore, these findings do not exclude the possibility that the SCFAs have a beneficial effect on SARS-CoV-2 infection, since in vivo such effects may be dependent on interactions with different cell types, and even if SCFAs were to have negative effect in regard to SARS-CoV-2 replication, it may be outweighed by their confirmed anti-inflammatory properties.

Notably, SCFAs also enter the brain through the blood–brain barrier and bind to the G protein-coupled receptors, especially FFAR2 and FFAR3, exerting influence on key neurological and behavioral processes in the brain [52]. Research on gut–brain axis have shown that gut microbiota dysbiosis is present in several neuropsychiatric disorders that include anxiety [53], depression [54], dementia [55], and neurosensory abnormalities of taste [56]—which according to some reports may be present in up to half of hospitalized COVID-19 patients [57]. Some researchers have pointed out that a COVID-19-related decrease in SCFA-producing bacteria resulting in SCFA deficiency may be associated with brain inflammation and disturbance in neuronal processes, which could explain the presence of neuropsychiatric complications of COVID-19[58].

### Application of short-chain-fatty acids in clinical setting

The increasing understanding of the role SCFAs play in both physiology and pathology has prompted some researchers to investigate the possibility of modifying their levels through indirect (probiotics or dietary fiber) or direct (SCFAs supplementation) means.

SCFAs can be administered in food or as tablets releasing SCFAs into the colon, or by enemas, though there is some confusion surrounding the systemic bioavailability of SCFAs acquired through the gut [27, 59]. Early studies have shown some promise in using butyrate/SCFA enemas to improve disease activity scores in patients with IBD and colitis, and its use has been reported for multiple conditions including ulcerative colitis, diversion colitis, radiation proctitis, and pouchitis, but later studies found no significant benefits to enema approach [60]**.** The studies reported suffer from the lack of a standardized protocol, and variation in concentration and frequency of the provided enemas, as well as use of saline enemas as placebo—which itself has a positive biological effect on the gut—have likely contributed to mixed results seen [61, 62].

An alternative to enemas, orally administered pH-dependent, slow-release butyrate tablets have shown some benefits in patients with diverticulosis and Crohn’s disease in pilot studies [63], but so far, this approach remains largely unexplored. In animals, orally delivered SCFAs appear to reduce systemic inflammation, intestinal permeability and have neuroprotective properties [30], in line with results suggested by basic research.

Interestingly, SCFAs can be found in routinely consumed food as well, such as cheese, butter, alcoholic beverages, vinegar, pickles, sauerkraut, soy sauce, and yoghurt [64–66]. For example, oral ingestion of vinegar results in rapid absorption of acetate and transient increase in circulating acetate levels [67]. Fermented food products reportedly improve health and prevent diseases, including cardio-metabolic disease and type 2 diabetes, but the underlying mechanisms have not been completely explored to date [68]. It is difficult to estimate how much SCFAs levels found in fermented food contribute to their health benefits, but it needs to be reiterated that the majority of SCFAs stem from microbial fermentation of dietary fiber and not from exogenous intake. Role of fermented foods consumption in regard to COVID-19 is also unknown, although some researchers pointed out that populations characterized by high consumption of fermented vegetables may have lower COVID-19 mortality due to anti-inflammatory and anti-oxidant properties of fermented foods [69], possibly owing to the myriad of compounds found in them, SCFAs included.

All mentioned studies support the statement that SCFAs and especially butyrate have beneficial and worth investigating anti-inflammatory and immunomodulatory functions that might be used to alleviate the course of the disease in COVID-19 patients. Unfortunately, to our knowledge, so far, no clinical study has examined the effects of SCFA supplementation in COVID-19 patients. A wide range of pre-clinical evidence supports the idea that SCFA supplementation may be beneficial, but until a sufficient number of human studies is conducted, its use cannot be recommended.

## Microbiota

### Gut microbiome

The gut microbiome is formed of trillions of diverse bacteria dwelling in the gut, which collectively exert a multitude of effects on regulatory mechanisms of immune response and metabolism. The gut microbiome is dynamic, and its composition is regulated not only by host genetic factors, but also by changing environmental and dietary factors [70]. Its makeup can also undergo modifications to facilitate a stimulatory or suppressive response to internal or external agents such as infectious pathogens [71].

Healthy person’s bacteria in bowel consist of mainly *Bacteroidetes* (e.g., *Bacteroides*) and *Firmicutes* (e.g., Lactobacillus*, Bacillus*, and *Clostridium*), with lower number of *Actinobacteria* (e.g., *Bifidobacterium*) and *Proteobacteria* (e.g., *Escherichia*) [72, 73]. These bacteria exist in a symbiotic relationship with its host, facilitating the synthesis of vitamins and fermentation of carbohydrates and other nutrients, as well as regulating mucosal permeability and immune response. Gut microbiota is also known to indirectly influence processes taking place at distant sites, acting through an extensive web of bidirectional intersystemic interactions, such as the still not fully explored lung–gut axis, of which SCFAs constitute a major integral part.

Dysbiosis, which may be defined as an imbalance in the normal composition of the microbiome, is commonly found in a variety of infectious and non-infectious diseases, often involving chronic inflammation or impaired metabolism, which include IBD, cardiovascular disease, and diabetes [74]. SARS-CoV2 infection alters the gut microbiota, causing a decrease in the diversity of the microbiota, increasing the number of opportunistic pathogens such as *Clostridium hathewayi*, *Actinomyces viscosus*, Streptococcus and *Veillonella* along with reduction in beneficial bacteria, including *Faecalibacterium prausnitzii*, which is one of the butyric acid-producing bacteria [75]. Abundance of *Coprobacillus, Clostridium ramosum,* and *Clostridium hathewayi* was positively correlated with COVID-19 severity, while abundance of SCFA-producing *Faecalibacterium prausnitzii* was inversely correlated with disease severity [76]. In a study involving 66 COVID-19 patients, fecal concentrations of SCFAs were significantly lower in patients with COVID-19 with severe/critical illness than that in non-COVID-19 controls, and observed simultaneous depletion of *F. prausnitzii* appears to contribute to this reduced capacity for SCFA biosynthesis in patients with COVID-19 [77]. In the same study, SCFA fecal levels remained decreased even at the final follow-up, 30 days after disease resolution, indicating that SARS-CoV-2 infection may have a long-lasting effect on gut microbiome composition.

The idea that disturbance in levels or composition of SCFA-producing bacteria may influence pathologic processes taking place both in the gut and other organs is upheld by observations in a number of both infectious and non-communicable diseases [78]. For example, a decrease in the butyrate-producing species *Roseburia hominis* and *Faecalibacterium prausnitzii* has been observed in patients with ulcerative colitis [79], while reduced levels of propionate producers are detected in children at risk of asthma [80]. Decreased levels of SCFAs due to depleted number of *Faecalibacterium* have even been linked to severity of disease and outcome in patients with encephalitis [81], showing a wide range of diseases with some connection to dysbiosis involving SCFA-producing bacteria.

Furthermore, not only SARS-CoV-2 infection results in gut dysbiosis, but also the existing dysbiosis itself contributes to the course and severity of COVID-19. Increased gut permeability as a result of dysbiosis can lead to pro-inflammatory bacterial products to leak out, triggering the release of pro-inflammatory cytokines and potentially further predisposing individuals to a “cytokine storm” in severe COVID-19 infection [82, 83]. This is an especially interesting observation in the context of elderly and immunocompromised populations, which are known to have reduced microbiota diversity, and at the same time have statistically worse clinical outcomes for COVID-19. It also further supports the idea that SCFAs, especially butyrate, could reduce the risk of cytokine storm in COVID-19 patients through inhibition of the pro-inflammatory NF-κB pathway, promotion of anti-inflammatory M2 macrophages and ability to improve epithelial barrier integrity [45].

Recently, long-term complications of COVID-19 and its’ commonness are being emphasized [84]. Post-acute COVID-19 syndrome (PACS) is increasingly worldwide recognized. According to Liu et al., 76% of patients are presented with at least on symptom at 6 months after recovery [85]. Moreover, authors stated that gut microbiota composition at admission was strongly linked with PACS occurrence. PACS-free patients showed recovered gut microbiome profile at 6 months in comparable to that of non-COVID-19 control patients. SCFAs producing bacteria, including *Bifidobacterium pseudocatenulatum* and *Faecalibacterium prausnitzii* were characterized with the largest negative correlations with PACS.

### Microbiome and the lungs

Until recently, the lung was thought to be a sterile site free from bacteria. In reality, the whole respiratory tract is a microbiome rich site, with diverse and dynamic bacterial flora. The lung microbiota is less dense and constitutes a significantly lower biomass than the gut microbiota, but the major bacterial phyla in the lungs are the same as in the gut, mainly *Firmicutes* and *Bacteroidetes* followed by *Proteobacteria* and *Actinobacteria* [86]. Lung microbiome is thought to be involved in a variety of infections and non-communicable diseases. For example, there is a relationship between lung microbiota and susceptibility to pneumonia, and the microbial composition appears to differ according to the causative agent of pneumonia [87]. Viral infections caused by rhinovirus, influenza virus, adenovirus, parainfluenza virus and RSV may result in lung dysbiosis as well [88, 89].

There is an intense interaction between processes taking place in the gut and the lungs, and the composition of the gut microbiota appears to contribute to pulmonary health and disease [90, 91], and vice versa. For example, several studies have shown that enterically administered probiotics may prevent upper respiratory tract infections [92]. There is research showing that modification of newborns' diet can influence the composition of their lung microbiota, and fecal transplantation in rats results in changes in the lung microbiota [93, 94]. Gut dysbiosis has also been linked to an increased risk of developing asthma later [95, 96]. On the other hand, some studies have suggested that respiratory viral infections may be associated with altered gut microbiome, which predisposes patients to secondary bacterial infections [88]**.** The exact mechanisms are not yet fully understood, but it might be partially explained by the fact that microbial components like endotoxins and metabolites (i.e. SCFAs) from the gut can contribute to the regulation of systemic immune responses; they may also diffuse into the systemic circulation and thus potentially exert an effect on distant organs. This relation may be bidirectional.

The lung is the main affected organ in COVID-19, but so far, the relation between the disease and the lung microbiota remains unknown. In a few available studies on the lung microbiome of COVID-19 patients, there are significant changes in its composition, but the number of tested individuals is by far too small to reliably draw any conclusions regarding the reported pathogen-enriched lung microbiota [97–99].

### Modulation of microbiota composition through application of probiotics in clinical setting

Having recognized the link between COVID-19 and gut microbiota dysbiosis, it is justified to ask oneself if there are potential benefits of using probiotics as adjunctive therapy in COVID-19. In fact, probiotics have previously shown good results in improving inflammatory conditions, regulating innate immunity and restoring gut barrier integrity [100], although it is difficult to estimate to which extent SCFAs alone are responsible for this effect. A recent systematic review showed that more than twenty probiotic bacteria strains improve the anti-inflammatory interleukins and anti-body production against various viruses, and virus titers were lowered after probiotics supplementation. Probiotic-containing foods and fermented food products also showed effect on prevention and treatment of viral diseases [101]. Studies have demonstrated that the immune modulation deriving from probiotic bacteria (i.e. specific strains of *Lactobacillus*) may be due to the release of the anti-inflammatory cytokines in the gut [102] or, in the case of *Bifidobacterium* species*,* modulation of the functional metabolism of T regulatory cells [103]. Furthermore, some probiotic strains such as *E. faecium* could significantly downregulate the mRNA level of TLR4, TLR5, TLR7, and TLR8, which are pattern recognition receptors pivotal in regulating the inflammatory response to a range of microbes, including viruses, in the GI tract. On the other hand, some probiotics can induce the production of INF, which is involved in pro-inflammatory response to viral infections. In vitro, *L. lactis* JCM5805 can activate human plasmacytoid dendritic cells, which play a crucial role in antiviral immunity as proficient type I IFN-producing cells [101]. *Bifidobacterium animalis* can inhibit the replication of SARS-COV-2 by reducing endoplasmic reticulum stress-related autophagy, especially through the Inositol-Requiring Enzyme 1 pathway, over its anti-interleukin-17 effect.

Supplementation of selected bacterial SCFA producers alone is an interesting concept, which has been considered in the context of IBD, and in CD-based in vitro simulations addition of butyrate-producing bacteria, especially *Butyrococcus pullicaecorum*, improved epithelial barrier integrity [104]. Probiotics containing *Lactobacillus plantarum, Lactobacillus acidophilus*, *Lactobacillus rhamnosus* and *Enterococcus faecium* have been found to increase production of SCFA by the intestinal microbiome [105].

In February, 2020, China's National Health Commission suggested the use of probiotics along with conventional treatment in patients with COVID-19 infection to improve the balance of intestinal flora and prevent secondary bacterial infections. This was met with skepticism from some researchers. They stressed that rationale for using probiotics in COVID-19 has been mostly derived from indirect evidence, and that up to date there is not enough clinical data regarding use of any probiotics in COVID-19 patients to recommend their implementation in regular treatment [106].

Adverse effects of probiotics appear to be mild (mainly digestive belching or bloating), but severe consequences (such as fungaemia and bacteremia) have been noticed in individuals in immunosuppression [107, 108]. Moreover, “probiotic” is a general term for a variety of pharmaceuticals, and depending on their composition, the effects of their intake may vary greatly.

## Conclusion

Long-standing research highlights the impact of nutrients and compounds found in food on immunological responses to various pathogens. SCFAs emerge as major mediators, which could link nutrition, gut microbiota, physiology and pathology. Many biological effects seem to be mediated by these bacterial metabolites, and a wide range of pre-clinical evidence supports the notion that their clinical use could potentially be very beneficial, but there is not enough clinical research data to back this claim. More research is still needed to explore the mechanisms of production and action of SCFA, and clinical tests need to be conducted to examine the extent to which they can be employed in prevention and treatment of diseases, including COVID-19.

## Materials and methods

In this review, search terms were selected to identify literature on the role of SCFAs in COVID-19 diagnosis, treatment and prognosis. Key terms used during research were “short chain fatty acids”, “coronavirus infection”, “COVID-19”, “coronavirus receptors”, and “microbiota”. Results were limited to relevant papers published in English. A total of 187 studies were retrieved, plus 30 studies derived from review articles. There were no restrictions on the publication date for the articles cited in all subsections of the manuscript. The first search was performed on January 30, 2021, and the search was updated on March 30, 2022, with a final revision on 13 August 2022. After screening and assessment for eligibility, finally a total of 20 studies were included.

## Data Availability

Not applicable.

## Abbreviations

ACE2: Angiotensin converting enzyme -2
ASGR1: Asialogycoprotein receptor 1
AXL: Tyrosine-protein kinase receptor UFO
GI: Gastrointestinal
HDAC: Histone deacetylase
IBD: Inflammatory bowel disease
IBS: Irritable bowel syndrome
KREMEN1: Kringle-containing protein marking the eye and the nose protein 1
PPAR: Peroxisome proliferator-activade receptor
SARS-CoV-2: Severe acute respiratory syndrome coronavirus 2
SCFA: Short-chain fatty acids
TLR: Toll-like receptor
